# Optimized Adaptive Local Iterative Filtering Algorithm Based on Permutation Entropy for Rolling Bearing Fault Diagnosis

**DOI:** 10.3390/e20120920

**Published:** 2018-12-01

**Authors:** Yong Lv, Yi Zhang, Cancan Yi

**Affiliations:** 1 Key Laboratory of Metallurgical Equipment and Control Technology, Wuhan University of Science and Technology, Ministry of Education, Wuhan 430081, China; 2 Hubei Key Laboratory of Mechanical Transmission and Manufacturing Engineering, Wuhan University of Science and Technology, Wuhan 430081, China

**Keywords:** adaptive local iterative filtering, particle swarm optimization, permutation entropy, fault diagnosis

## Abstract

The characteristics of the early fault signal of the rolling bearing are weak and this leads to difficulties in feature extraction. In order to diagnose and identify the fault feature from the bearing vibration signal, an adaptive local iterative filter decomposition method based on permutation entropy is proposed in this paper. As a new time-frequency analysis method, the adaptive local iterative filtering overcomes two main problems of mode decomposition, comparing traditional methods: modal aliasing and the number of components is uncertain. However, there are still some problems in adaptive local iterative filtering, mainly the selection of threshold parameters and the number of components. In this paper, an improved adaptive local iterative filtering algorithm based on particle swarm optimization and permutation entropy is proposed. Firstly, particle swarm optimization is applied to select threshold parameters and the number of components in ALIF. Then, permutation entropy is used to evaluate the mode components we desire. In order to verify the effectiveness of the proposed method, the numerical simulation and experimental data of bearing failure are analyzed.

## 1. Introduction

Rolling bearing is one of the parts of rotating machinery, which are widely used and easily damaged in mechanical equipment [1,2,3,4]. The running state of rolling bearings will directly affect the production efficiency and safety of the equipment. In the actual operation of the mechanical equipment, if the early fault of the rolling bearing cannot be identified in time, the impact of the fault will accelerate the damage of the rolling bearing and lead to the failure eventually, which has a serious effect on the regular production [5,6,7,8]. Therefore, it is very important to monitor and diagnose the rolling bearing’s running state, especially for the early fault [9,10].

The analysis of the vibration signal is an important approach for monitoring the running state of mechanical equipment and the particularly significant task is to extract the fault feature frequency from the complex vibration signal accurately [11,12,13]. For the early failure of mechanical system, it is in the germination stage and the difference between its performance and normal state is small. Consequently, it is often expressed as a weak signal of instability [8]. Additionally, the early weak fault signal is inundated in the strong background noise, which presents a complex non-stationary and nonlinear characteristic. Thus, it is more difficult to inspect the weak signal of the early fault directly [14,15,16,17,18]. Recently, a large number signal processing method has been studied, such as Short Time Fourier Transform (STFT), Wavelet Transform (WT) and Empirical Mode Decomposition (EMD). The basic idea of STFT [19,20] was to assume that the non-stationary signal was stationary in a short interval within a given analysis window. It was only suitable for the analysis of slow varying signals and it was difficult to obtain better performance for the widespread frequency fast changing signals in nature. WT [21,22,23,24] combined the characteristics of the trigonometric function, the wavelet basis function and the time-lapse window function in the STFT and formed a new vibration and attenuation basis function, which had a good local characteristic. However, the wavelet transform was essentially a window-tunable Fourier transform that did not get rid of the limitations of the Fourier transform and the wavelet basis was difficult to select, which lacks of adaptability. To get rid of the limitation of the above time-frequency analysis method and obtain the desire performance of time-frequency analysis. In 1998, N. E. Huang of NASA proposed EMD [25,26], which was based on the concept of instantaneous frequency estimation. The empirical mode decomposition can decompose a complex signal into the sum of the finite intrinsic modal functions (IMFs) adaptively. Each intrinsic mode function (IMF) component represents a set of characteristic scale signals. Therefore, the energy feature extraction of each component can better reveal the inherent characteristics of fault information. However, this method produces modal aliasing and the number of components is uncertain, which leads to the unsatisfied performance in decomposing different orders in classification and feature identification.

Recently, Cicone et al. has proposed a new adaptive time-frequency analysis method, namely adaptive local iterative filtering (ALIF), which can be used to deal with nonlinear and non-stationary signals [27,28]. ALIF is an adaptive decomposition method based on iterative filtering (IF). The difference between the two methods is that ALIF realizes adaptive decomposition of signals by choosing the length of the filters adaptively. Moreover, ALIF can avoid the modal aliasing effectively and decompose the signals with same scale and order. Significantly, it overcomes the shortcomings of EMD’s lack of theoretical basis and noise sensitivity. According to the principle of ALIF algorithm, the result decomposed by ALIF which is limited by the selection of threshold parameters and the number of components. In order to extract the weak fault information, the selection of parameters need to be optimized. Considering the fast convergence speed, small setting parameters and easy realization of the particle swarm optimization (PSO) [29,30,31], it is applied to the parameter optimization in ALIF. The original signal is decomposed into a series of components by the improved ALIF method and the optimal component is identified by the principle of permutation entropy [32,33,34,35]. Theoretically, the value of the permutation entropy can reflect the complexity of the signal, so as to select the optimal component. The advantage of this improved method is that the parameter selection and decomposition results of ALIF algorithm are not affected by human experience. 

As an early fault of a bearing to extract the early fault from the vibration signal with low signal-to-noise ratio. The spectral kurtosis proposed by Dwyer [36] can indicate how the impulsiveness of a signal with frequency. Antoni proposed Fast Kurtogram (FK) [37] and proved its effectiveness in early fault diagnosis [38,39]. However, FK has several disadvantages, especially when the noise is strong [40]. The enhanced kurtogram proposed by Wang D [41] can help to locate the resonance frequency band for further demodulation by envelope analysis. However, in many cases, discrete frequency noise always exists and may cover up weak bearing faults. Lei Y. proposed an improved kurtogram method by introducing WPT into kurtogram, in which WPT as the filter of kurtogram to overcome the shortcomings of the original kurtogram [42]. Tse P. W. proposed a new concept called sparsogram and the purpose of sparsogram is to quickly determine the resonant frequency band. The sparse graph is constructed by measuring the sparsity of the power spectrum of the envelope of the wavelet packet coefficients at different wavelet packet decomposition depths [43]. It can be seen that the enhanced kurtogram and sparsogram aim to characterize the cyclostationality of bearing fault signals, which significantly improves the fast kurtogram and the improved kurtogram. This is because the improved kurtogram and the fast kurtogram are sensitive to impulsive noises and they cannot be used to characterize the cyclostationarity of bearing fault signals. Due to the early fault of rolling bearings is weak and submerged by noise, these shortcomings limit their application in fault diagnosis of rolling bearings.

In this paper, an improved ALIF decomposition method is proposed, which joins the particle swarm optimization (PSO) and permutation entropy (PE) to select the optimal components. The threshold parameters and number of components in ALIF are usually chosen by artificial experience. What is more, parameters selection based on experience are often inaccurate. Therefore, this paper proposes an optimized adaptive local iterative filtering based on particle swarm optimization. Bandt and Pompe proposed the concept of mean entropy parameter in measuring the complexity of one-dimensional time series, which is called PE [44]. This is an index used to describe the irregular or nonlinear systems. It is difficult to describe quantitatively. PE has the advantages of simple calculation, strong anti-noise and high sensitivity to signal changes. It can also be applied to weak signal detection and dynamic mutation of complex systems. When gear and bearing parts are running malfunction or different types of faults, the influence of nonlinear factors and signal complexity is different. Therefore, the vibration signals obtained from the mechanical system will also change, resulting in different values of PE. Subsequently, PE is used as a new method to select the optimal components needed for reconfiguration. To verify the effectiveness of the proposed method, the bearing fault vibration signal measured from test-bed is used for the result evaluation. Furthermore, this method is also compared with the EMD.

This paper was organized as follows: the basic principle and characteristics of feature extraction for bearing fault based on Optimized adaptive local iterative filtering and permutation entropy (PE) are introduced in the Section 2. In Section 3, a numerical simulation experiment is carried out to verify the effectiveness of the proposed algorithm. In Section 4, the effectiveness of the proposed method is verified by the application of bearing data in the intelligent maintenance system center of the University of Cincinnati. The conclusions of the study and the necessary discussions are given in Section 5.

## 2. The Theory of the Proposed Method

### 2.1. The Theory of Adaptive Local Iterative Filtering

The adaptive local iterative filtering (ALIF) method is the improved version of iterative filtering [45,46,47,48]. The main difference between the two methods is that the former can locally and adaptively calculate the length of filters and generate a moving average line using the solution of Fokker Planck equation, which is so-called FP filter. Adaptive local iterative filtering algorithm is listed in Algorithm 1.

Given a signal f(x) and x∈R, we define $L_{w_{n},l_{n}}(f)(x)=\int_{-l_{n}(x)}^{l_{n}(x)}f_{n}(x+t)w_{n}(x,t)dt$, which represents the moving average function of the signal f(x). In the above function, $w_{n}(x,t)$ is a filter represented by step n and the length is $2l_{n}(x)$, in which $t\in [-l_{n}(x),l_{n}(x)]$. $w_{n}(x,t)$ indicates that $w_{n}$ changes with x. According to the definition of the moving average, we can also define an operator $S_{1,n}(f_{n})=f_{n}-L_{w_{n},l_{n}}^{\left(1\right)}(f_{n})=f_{n+1}$ to capture the fluctuation of $f_{n}$, in which the superscript reflects the number of IMF, thus the first IMF can be represented as $I_{1}=\lim_{n\to \infty }S_{1,n}(f_{n})$.

There are two loops, an inner loop and an outer loop in the adaptive local iterative filtering algorithm. The inner loop is to capture a single IMF and the outer loop is to generate all the IMFs of the original signal. The iteration equation of the inner loop is defined as follows:

(1) $$f_{n+1}(x)=f_{n}(x)-\int_{-l_{n}(x)}^{l_{n}(x)}f_{n}(x+t)w_{n}(x,t)dt$$

The $l_{n}(x)$ in the formula is filtered length and can be obtained by the following formula in IF:

(2) $$l_{n}(x)=2[v\frac{N}{k}]$$

where v is set at 1.6, k is the number of extreme points and N is the length of original signal.

**Algorithm 1** Adaptive local iterative filtering algorithmALIF Algorithm IMF = ALIF(f)IMF = { } **While** the number of extrema of f≥2
**do** $f_{1}=f$ **While** the stopping criterion is not satisfied **do** Compute the filter length $l_{n}(x)$ for $f_{n}(x)$ $f_{n+1}(x)=f_{n}(x)-\int_{-l_{n}(x)}^{l_{n}(x)}f_{n}(x+t)w_{n}(x,t)dt$ n=n+1 **End while** $IMF=IMF\cup \{f_{n}\}$ **End while** IMF=IMF∪{f}

According to the following formula, it is possible to determine if the I component satisfies the IMF condition:

(3) $$SD=\frac{||I_{1,n}-I_{1,n-1}|{|}_{2}}{||I_{1,n-1}|{|}_{2}}$$

When the SD in the formula is less than the set value, the extracted component is IMF.

When a given signal shows strong non-linear and non-stationary characteristics, the smoothness of the IMF component decomposed by IF algorithm with fixed filter function cannot be guaranteed. It is necessary to solve the filtering function adaptively in the corresponding filtering interval to obtain the smoothness of the IMF component. Cicone et al. constructed FP filter function by using Fokker-Planck differential equation basic solution system and realized adaptive adjustment of filter function in iterative filter decomposition process.

If there are two derivable functions g(x) and h(x), it is satisfied on a<0<b as follows:

(1) g(a)=g(b)=0 and g(t)>0 for x∈(a,b)
(2) h(a)<0<h(b)

The general form of Fokker-Planck equation is

(4) $$\frac{\partial p}{\partial t}=-\alpha \frac{\partial (h(x)p)}{\partial x}+\beta \frac{\partial^{2}(g^{2}(x)p)}{\partial x^{2}}$$

where α and β in the formula is the steady-state coefficient in the range of (0,1).

In the equation $\frac{\partial^{2}(g^{2}(x)p)}{\partial x^{2}}$ produces diffusion effect and drives the solution p(x) to move from the center of the interval (a,b) to the end point a and point b; Meanwhile, $-\alpha \frac{\partial (h(x)p)}{\partial x}$ makes p(x) converge from both ends of point a and point b to the center of the interval (a,b). When the two are balanced, there are as follows:

(5) $$-\alpha \frac{\partial (h(x)p)}{\partial x}+\beta \frac{\partial^{2}(g^{2}(x)p)}{\partial x^{2}}=0$$

At this point, the equation has a non-zero solution p(x) and satisfies the condition:

(1) p(x)>0, for any x∈(a,b)
(2) p(x)=0, for any x∉(a,b)

This means that the solution set of the equation falls on the interval (a,b), then the solution of the Fokker-Planck equation p(x) is the filter function w(t). w(t) has different expressions within the changes of the interval (a,b) and we let interval (a,b) substitutes for mask length $\left(-l_{n}(x),l_{n}(x)\right)$, thus realizing the adaptive solution of filter function by ALIF method.

### 2.2. Adaptive Local Iterative Filtering Based on Particle Swarm Optimization

In the traditional adaptive local iterative filtering decomposition algorithm, the user needs to set the number of penalty parameters and components before the signal processing, due to the theoretical limitation of the existing algorithm. From the theoretical study of ALIF, it can be seen that when the threshold parameter is large, the bandwidth of each component after signal decomposition is large and vice versa. The number of components decomposed will also lead to different decomposition results. Therefore, how to choose the appropriate combination of parameters in the process of changing the number of components and threshold parameters to make the decomposed components match the input vibration signal is the key to obtain the fault information accurately.

Particle Swarm Optimization (PSO) is the most widely used intelligent optimization algorithm. Compared with other optimization algorithms, such as genetic algorithm and artificial fish algorithm, PSO has simple principle and mechanism, fast convergence of operation process, good convergence effect and global search performance [29,30]. Conclusively, it is suitable for the optimization of parameters in this article. Another key of the adaptive local iterative filter decomposition based on PSO scheme is the selection of fitness function. Fitness function is used to evaluate the quality of parameters in the process of parameter optimization in particle swarm optimization. It searches for the global optimal solution by following the current optimal solution. Here, the effect after decomposition is measured by evaluating the similarity between each model component and the original signal. Each decomposed model component and the original signal similarity can be expressed by Formula (6):

(6) $$C=\frac{\sum_{n=1}^{T}\left(r\left(n\right)-\bar{r}\right)\left(y\left(n\right)-\bar{y}\right)}{{\left[\sum_{n=1}^{T}{\left(r\left(n\right)-\bar{r}\right)}^{2}\sum_{n=1}^{T}{\left(y\left(n\right)-\bar{y}\right)}^{2}\right]}^{1/2}}$$

where the r(n),y(n) in the formula represents the original signal and the mode component respectively; $\bar{r},\bar{y}$ in the formula represents the average value of the original signal and the mode component respectively; T is denoted as the data length and C represents the cross correlation coefficient.

In the process of actual calculation and analysis, there may be a large fluctuation in the similarity between the decomposed model component and the original signal. In other words, the maximum of the average of the similarity does not necessarily mean the optimal result of the decomposition. In order to make the multi-mode components have a large cross-correlation coefficient, we introduce the mean and variance to evaluate it. The greater the mean value, the better the correlation between the pattern components and the original signal. Similarity, the smaller the variance, the smaller the deviations from the mean value. Therefore, the ratio of mean to variance of correlation coefficient can be used as fitness function to obtain the optimal threshold parameters and the number of components. The formula for fitness function is as follows:

(7) $$\mathrm{fitness}\;\mathrm{function}=\frac{\mathrm{mean}(C)}{\mathrm{var}(C)}$$

Based on the above theoretical analysis, the ALIF algorithm based on PSO is applied to the simulation signal analysis of rolling bearing and fault feature extraction of experimental system, which verifies the effectiveness of the method in fault diagnosis.

### 2.3. The Desired Component Selection Base on PE

After the optimization of the adaptive local iterative filter decomposition, it is necessary to select the optimal mode component in the decomposed component, which is very likely to be the component we need. The permutation entropy is a method to measure the uncertainty of one dimensional time series. Compared with the Lyapunov exponent and fractal dimension, its calculation is simple, the anti-interference effect is good and it can describe the small change of the sequence [49,50]. For the time series {x(t),t=1,2,…,N} with a length of *N*, the phase space reconstruction is carried out as follows:

(8) $$\{\begin{array}{c} X(1)=\{x(1),x(1+\lambda ),\ldots ,x(1+(m-1)\lambda )\}\\ X(i)=\{x(i),x(i+\lambda ),\ldots ,x(i+(m-1)\lambda )\}\\ X(N-(m-1)\lambda )=\{x(N-(m-1)\lambda ),x(N-(m-2)\lambda ),\ldots ,x(N)\} \end{array}$$

Among them, the embedding dimension is m and the delay time is λ. Rearrange all elements in X(t) in ascending order, that is $x(t+(j_{1}-1)\lambda )\leq x(t+(j_{2}-1)\lambda )\leq \ldots \leq x(t+(j_{m}-1)\lambda )$. The sequence X(t) can get the symbol sequence S(g)

(9) $$S(g)=\{j_{1},j_{2},\ldots ,j_{m}\}$$

Among them g=1,2,…k,k≤m!. There are altogether m! species of different symbol sequences S(g) in m dimensional phase space mapping. $p_{i}$ is defined as the probability of occurrence of the i symbol sequence and the entropy of the time series X(t) is

(10) $$H_{p}(m)=-\sum_{i=1}^{k}p_{i}\ln p_{i}$$

When $p_{i}=1/m!$, $H_{p}$ takes the maximum value of the ln(m!). Therefore, $H_{p}$ can be normalized as follows:

(11) $$H_{p}=H_{p}(m,\tau )/\ln(m!)$$

**Figure 1:**
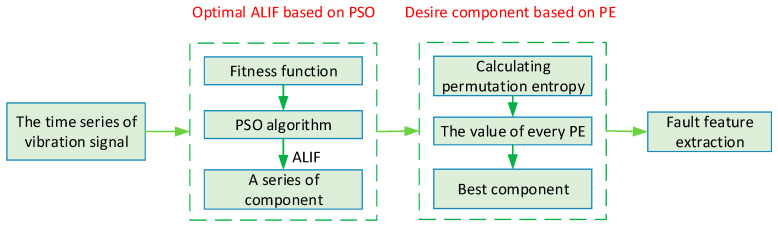
The flowchart of the proposed method.

Obviously $0\leq H_{p}\leq 1$. Permutation entropy can be used to measure the uncertainty and complexity of time series. The larger the value, the more complex the time series signal. Conversely, it indicates that the time series signal is simpler and more regular. Generally speaking, the entropy of arrangement of harmonic signals and modulated signals is relatively small, while the random noise’s permutation entropy is large. Therefore, permutation entropy can be used to select the optimal mode component for signal reconstruction. Finally, the flowchart of the proposed method is illustrated in Figure 1.

## 3. Numerical Simulation Analysis

**Figure 2:**
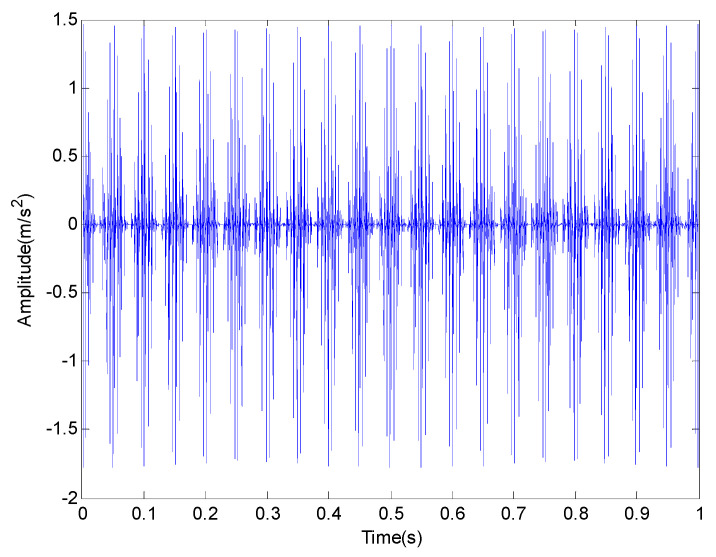
The original signal in time domain without noise.

**Figure 3:**
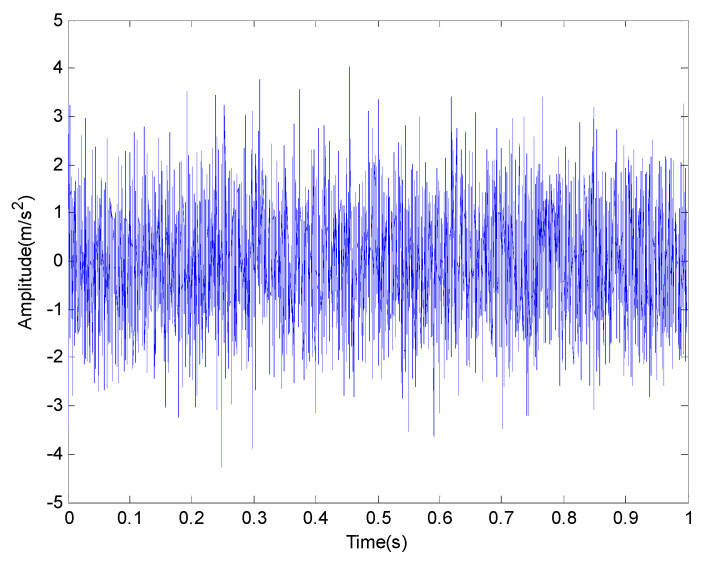
The original signal in time domain with noise.

**Figure 4:**
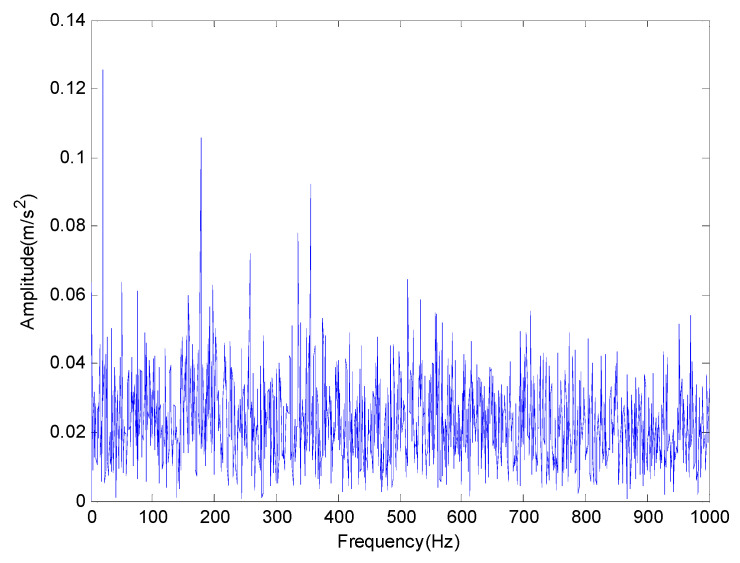
The simulated signal with noise in frequency domain.

Rolling bearings are mainly used in the rotating parts of mechanical equipment. The fault signals generally have characteristics typical of nonlinear and non-stationary and contain a large number of fault components and noise components. Extracting fault features from these signals is the key to fault diagnosis. There are many simulation models for bearing failures and the most classical one is proposed by Randall [51,52]. The fault simulation signal is as follows:

(12) $$\{\begin{array}{l} x(t)=s(t)+n(t)=\sum_{i}A_{i}h(t-iT-t_{i})+n(t)\\ h(t)=\exp(-Ct)\cos(2\pi f_{n}t+\varphi_{W})\\ A_{i}=C_{A}+A_{0}\cos(2\pi f_{r}t+\varphi_{A}) \end{array}$$

where s(t) is a periodic impact component; $A_{0}$ is the amplitude of resonance; $f_{r}$ is a modulation frequency; $\varphi_{A}$, $\varphi_{W}$ and $C_{A}$ can be arbitrary constant; C is a attenuation coefficient; T is defined as the average time between two shocks and $T=1/f_{p}$; $f_{p}$ is a fault characteristic frequency; $f_{n}$ is the resonance frequency of the bearing system and n(t) is the white noise component of Gauss. There are three common faults of rolling bearing: outer race fault, inner race fault and rolling element fault. In order to simulate these three kinds of faults, the modulation frequency can be set as $f_{r}=0$, $f_{r}=f_{r}$ and $f_{r}=f_{re}$ respectively. It should be pointed out that $f_{r}$ is rotational frequency and $f_{re}$ is retainer frequency. The outer race fault frequency $f_{o}$, the inner ring fault frequency $f_{i}$ and the rolling element fault frequency $f_{ro}$ are given in Table 1. Firstly, the feature extraction of inner race fault in strong noise environment is studied and some of the parameters are selected as shown in Table 2. In order to get closer to the actual situation, we add a Gauss white noise with a variance of 0.5. The sampling frequency and sampling points are set to 4096 Hz and 4096 points respectively. The original signal and the one with additive noise are shown in Figure 2 and Figure 3 respectively. The frequency spectrum is shown in Figure 4.

**Figure 5:**
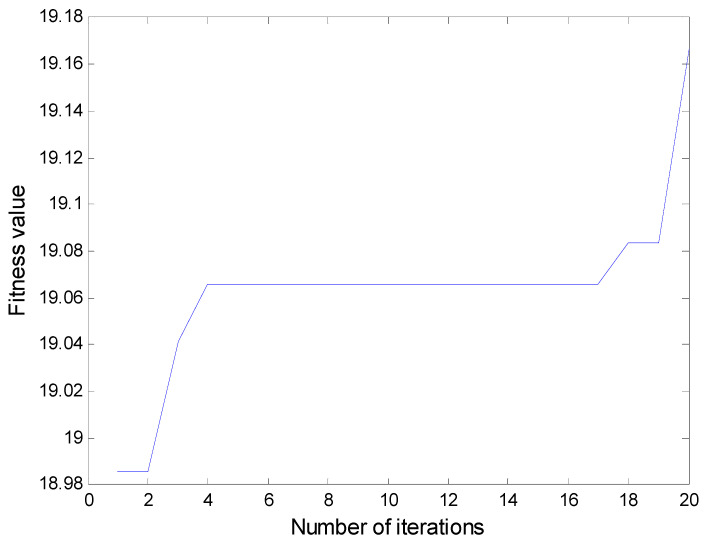
The fitness value during the iteration process.

In all numerical simulations and experimental studies, in order to keep a lower computational complexity, the delay time is usually chosen to be 1 in the calculation of PE. In addition, the threshold parameters and the number of components are obtained from the particle swarm optimization algorithm introduced in the Section 2.2. The number of iterations in the algorithm is set as 50 and the population size is determined as 20. The fitness value during the iteration process is plotted in Figure 5. The threshold parameters and the number of components are set as $7\times 10^{-5}$ and 4 respectively. According to Figure 3, the inner race fault characteristics of the original signal with noise in time domain cannot be clearly indicated in strong background noise. The result of the FFT analysis is shown in Figure 4, the frequency of the inner race fault is still obscured by noise components.

**Figure 6:**
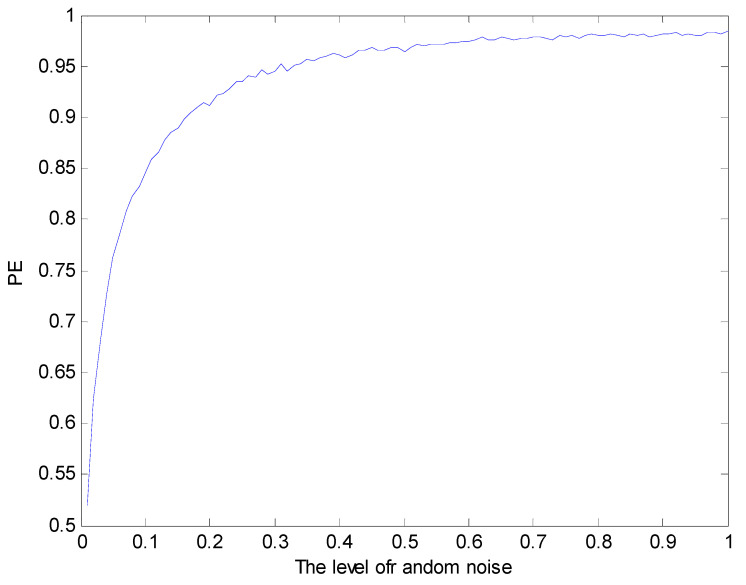
The value of permutation entropy (PE) varies with noise.

To illustrate the effectiveness of PE in evaluating the whole life cycle of rolling bearings, the method adopted here is to simulate the failure of rolling bearings from the beginning to the end. The equivalent method we use is to simulate the damage of bearings with different degrees of noise. The result is shown in Figure 6. It can be seen from Figure 6 that the value of PE is valid between 0.521 and 0.984.

**Figure 7:**
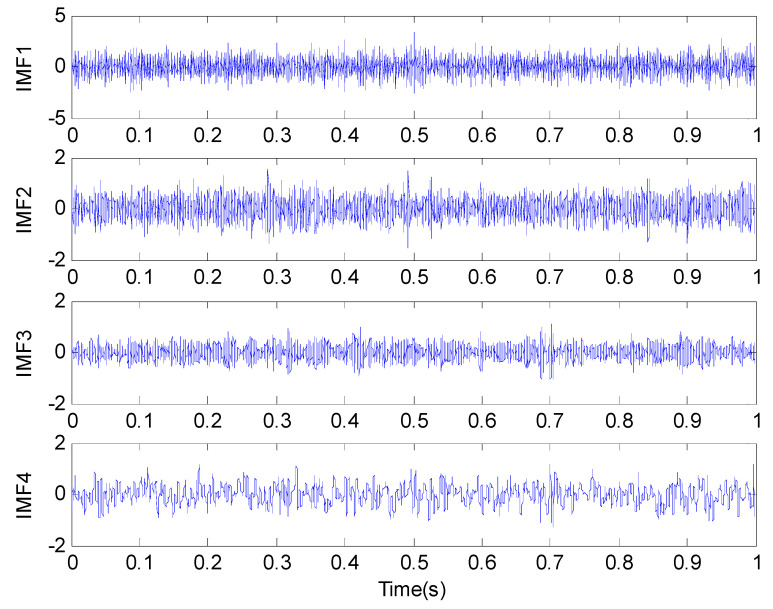
Decomposition results of the simulation signal provided by the proposed method.

The simulation signal is analyzed by the method proposed in this paper. After the original signal is processed by ALIF, it is decomposed into four IMFs adaptively as shown in Figure 7. In order to extract the components containing rich fault feature information from the results, each component of permutation entropy are calculated respectively as shown in Table 3, which IMF1’s permutation entropy is the largest, so to be identified as the best component.

**Figure 8:**
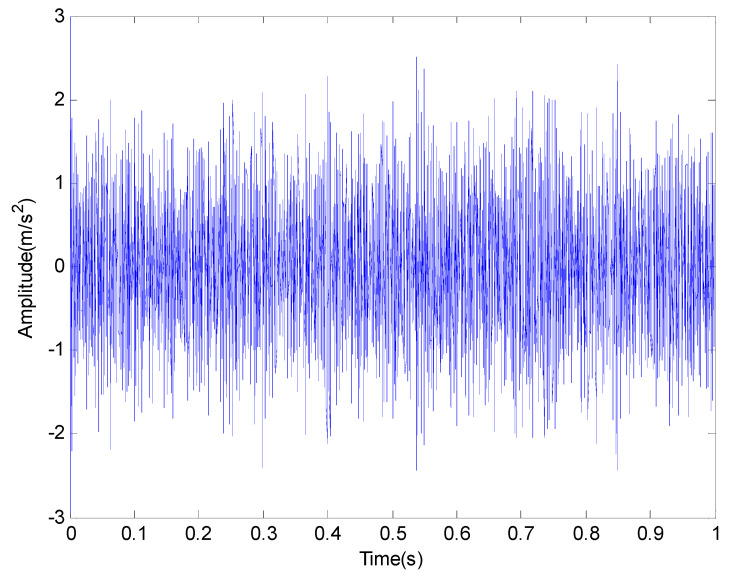
The best intrinsic modal function (IMF) of the original signal decomposed by empirical mode decomposition (EMD) in time-domain.

**Figure 9:**
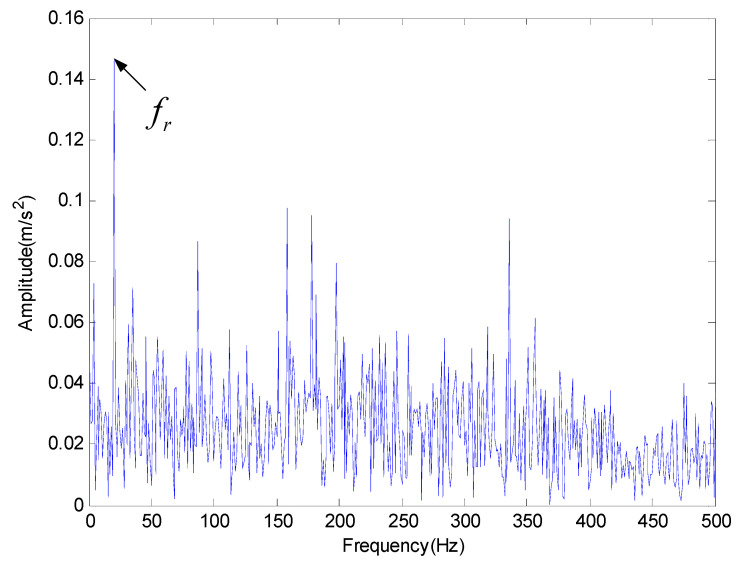
The simulation signal with noise in frequency domain.

**Figure 10:**
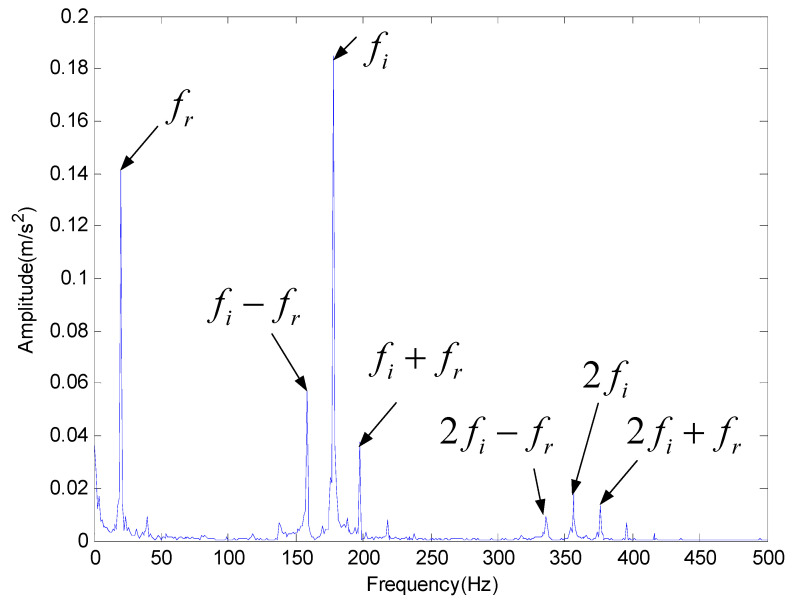
The analysis result provided by the proposed method for inner race fault.

For the simulation signal analysis, in order to evaluate the signal reconstruction of the proposed method under the noise condition accurately, the classical EMD method is used for comparison and analysis. Figure 8 is the best IMF of original signals decomposed by EMD in time-domain through cross correlation calculation. Figure 9 is the result of FFT analysis provided by EMD and only the rotating frequency ($f_{r}$) can be seen. Obviously, EMD cannot fully identify the characteristic frequency of inner fault. Figure 10 is the result provided by the proposed method based on PE in the frequency domain, which indicates the inner race feature frequency $f_{i}$ and its second harmonic frequency $2f_{i}$ can be identified.

**Figure 11:**
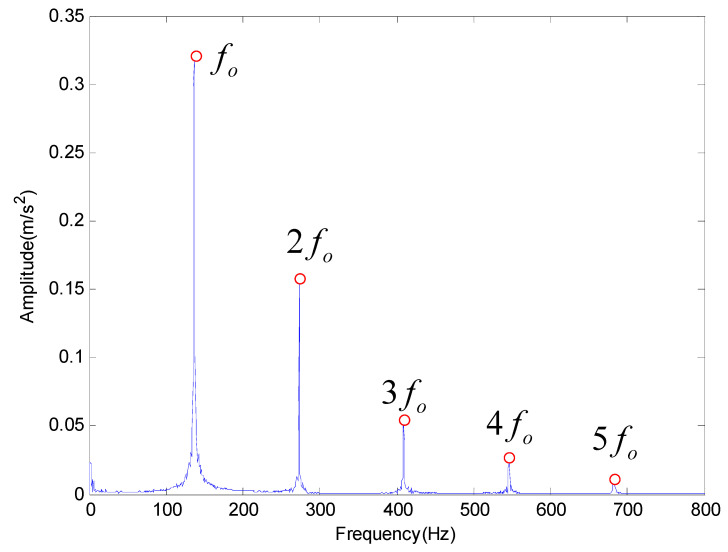
The analysis result provided by the proposed method for outer race fault.

**Figure 12:**
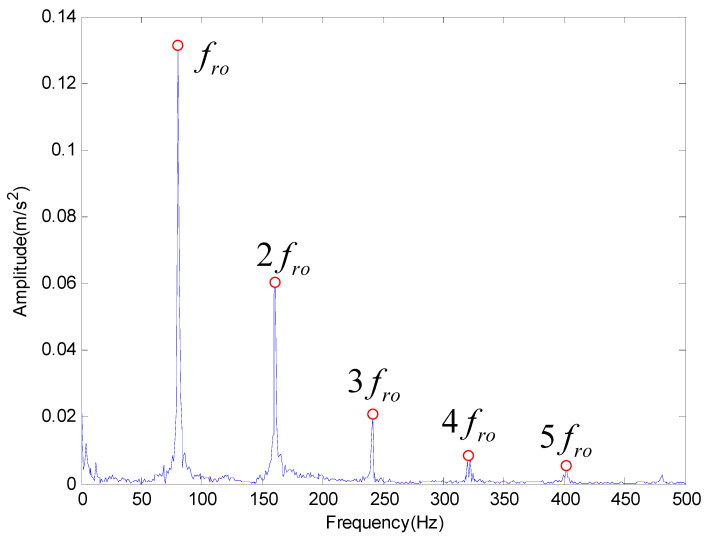
The analysis result provided by the proposed method for rolling element fault.

Then the proposed method is applied to extract the characteristic frequency of outer race and rolling element fault. The results obtained by the proposed method are shown in Figure 11 and Figure 12 respectively, which also can get the fault characteristic frequency and its frequency multiplication. Therefore, we can conclude that the proposed method has better performance in feature frequency extraction.

From the above analysis, it can be seen that the analysis of EMD is inundated in the frequency domain, which lead to the noise component and the original signal cannot be divided better. However, the optimized ALIF algorithm based on permutation entropy is more effective in feature frequency extraction and better reflects the fault characteristics. Through multiple simulation analysis, the optimized adaptive local iterative filtering based on permutation entropy is superior to the EMD in feature frequency extraction of rolling bearing.

## 4. Experimental Study

**Figure 13:**
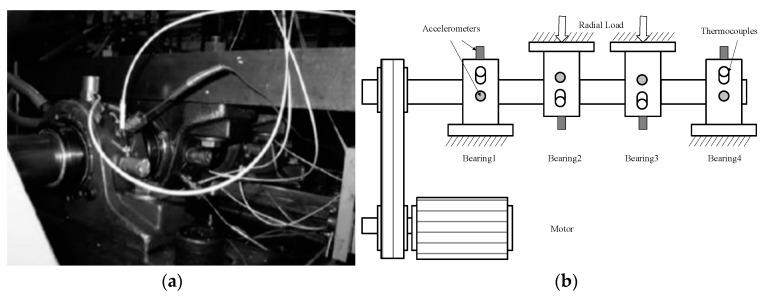
The Intelligent Maintenance System of Cincinnati: (**a**) Bearing test rig; (**b**) sensor placement illustration.

As an important part of rotating machinery, rolling bearing will have an important impact on production if it fails during operation. At present, the identification of rolling bearing fault still depends on the extraction of the characteristic frequency of the bearing fault. Analysis of the rolling life-cycle accelerated test data of the NSFI/UCR intelligent maintenance system center [53]. Four bearings are installed on the rotating shaft of the test bench at a rotational speed of 2000 r/min. Each bearing is equipped with an acceleration sensor in its axial and radial directions. Sampling frequency was set up to 20 kHz. The type of the acquisition card is 6062F of the American NI company. The mounting positions of the bearings and sensors are shown in Figure 13. The type of fault bearing is ZA2115 and its specific parameters are shown in Table 4. Three groups of all life experiments were performed and the information of three groups of data are shown as Table 5.

It should point out here that the calculated inner race fault frequency of rolling bearing is corresponding to $f_{o}$ = 223.17 Hz, the rotation frequency is $f_{r}$ = 33.33 Hz.

**Figure 14:**
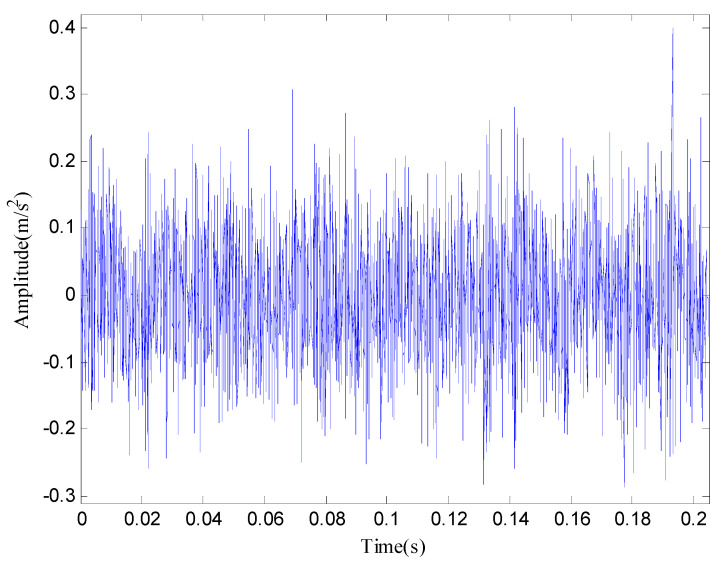
The original vibration signal in time domain.

**Figure 15:**
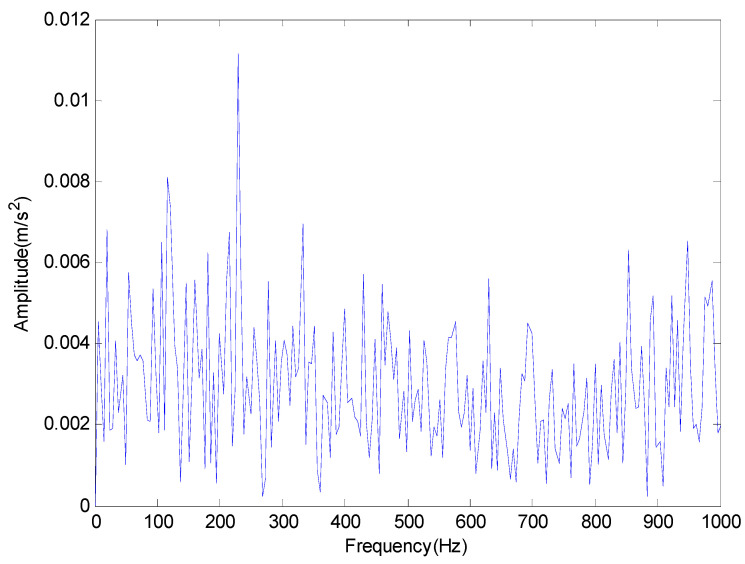
The original vibration signal in frequency domain.

The signal of rolling bearing 1 in the group 2 is used for analysis in this paper. Then, the time domain waveform and frequency domain diagram of the original signal are shown in Figure 14 and Figure 15 respectively.

**Figure 16:**
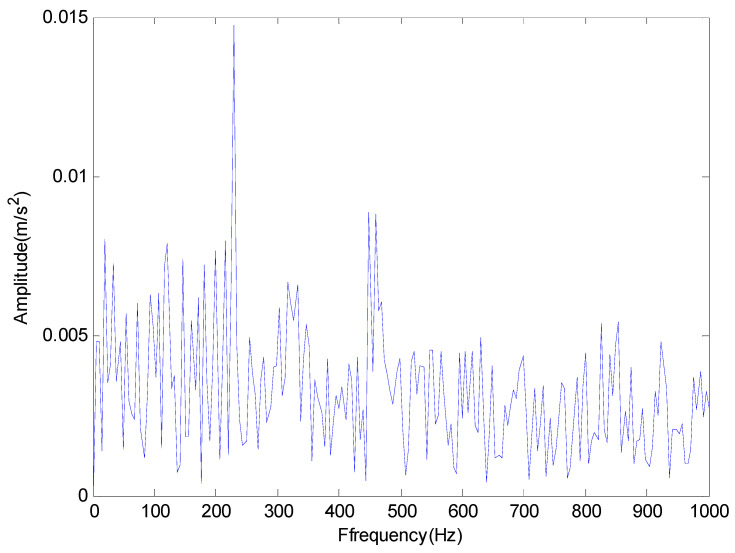
The result provided by EMD.

**Figure 17:**
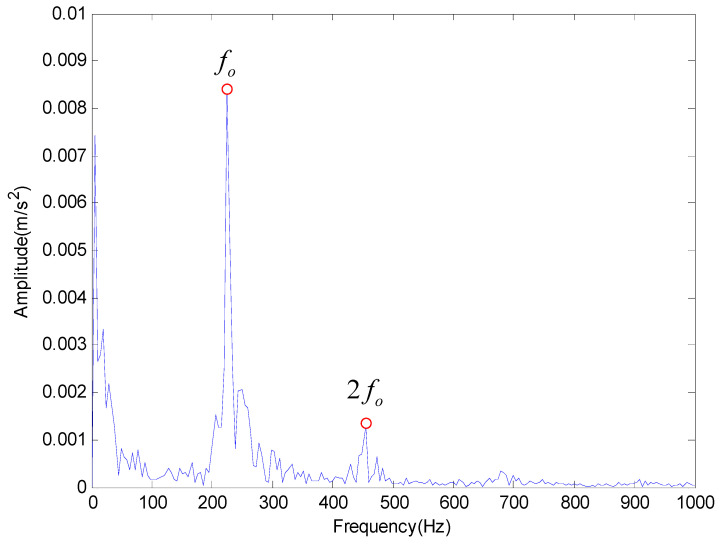
The result provided by the proposed method.

From the time domain waveform of Figure 14 and the frequency domain diagram of Figure 15, we cannot determine the location of the failure. Meanwhile, in order to illustrate the effectiveness of the proposed method, we compare the EMD with the proposed method. The results are shown in Figure 16 and Figure 17 respectively.

Then EMD was used to decompose the vibration signals and the result was plotted in Figure 16. It can be seen from the Figure 16 that the characteristic frequency is basically drowned by noise components after EMD. Subsequently, the method proposed in this paper is applied to decompose the vibration signals and the results are shown in Figure 17. Obviously, the outer race fault frequency $f_{o}$ and its resonant frequency $2f_{o}$ can all be easily identified in Figure 17. Therefore, we can draw a conclusion that the outer race of the rolling bearing is faulty, which is consistent with the actual situation.

## 5. Conclusions

This paper optimizes the adaptive local iterative filtering (ALIF) algorithm to solve the problem that its signal decomposition performance is largely depends on parameters selection. Moreover, the selection process lacks in self-adaptability. The main work of this paper is presented as follows: (1) the selection of threshold parameter and the number of components are performed by PSO using the mean and variance ratio of the correlation number as the fitness function; (2) the desired mode components selection is determined by using PE to improve the accuracy of the characteristic frequency; (3) The simulation and experimental data demonstrates that optimized adaptive local iterative filtering method based on PE has obvious superiority is more advantageous than other methods in the detection of the fault signal of rolling bearings, which shows that the method is feasible in practical application. Considering the development of data information fusion technology, what we need to do next is to extend the adaptive local iterative filtering to the processing of multi-channel signals. Multiple sensors contain more fault features, so it is easier to extract fault features.

## Figures and Tables

**Table 1:** Three kinds of fault characteristic frequency of rolling bearing.

Inner Race Frequency $f_{i}$ (Hz)	Outer Race Frequency $f_{o}$ (Hz)	Rolling Element Frequency $f_{o}$ (Hz)
180	135	80

**Table 2:** Parameter selection of inner race fault simulation signal.

$A_{0}$	$f_{r}$ (Hz)	$f_{m}$ (Hz)	$f_{n}$ (Hz)	C	$t_{i}$	$C_{A}$	$\varphi_{A}$	$\varphi_{W}$
3	20	20	2048	800	0.01	1	0	0

**Table 3:** The computed permutation entropy of each IMF component.

**IMF Order**	1	2	3	4
**PE Value**	0.8887	0.7072	0.5184	0.3754

**Table 4:** Structure parameters of rolling bearing.

Bearing Type	Outside Diameter d^2^/mm	Ball Diameter/mm	Ball Number n	Contact Angle α
**ZA2115**	71.5	8.4	16	15.17

**Table 5:** Description of three groups of bearing fault signal.

Group	Sample Channels	Fault Description
Group 1	8	Inner race defect that occurred in bearing 3 and roller element defect occurred in bearing 4.
Group 2	4	Outer race failure that occurred in bearing 1.
Group 3	4	Outer race failure that occurred in bearing 3.
